# Disease phenotype affects treatment of late-onset inflammatory bowel disease: Analysis of a large UK cohort

**DOI:** 10.1093/ibd/izag072

**Published:** 2026-05-08

**Authors:** Peter F G Foulser, Laura Martinez-Gili, Sharmili Balarajah, Rohan Sundramoorthi, James L Alexander, Benjamin H Mullish, Julian R Marchesi, Miles Parkes, Timothy R Orchard, Lucy C Hicks, Horace R T Williams, UK IBD BioResource Investigators, Peter F G Foulser, Laura Martinez-Gili, Sharmili Balarajah, Rohan Sundramoorthi, James L Alexander, Benjamin H Mullish, Julian R Marchesi, Miles Parkes, Timothy R Orchard, Lucy C Hicks, Horace R T Williams

**Affiliations:** Department of Metabolism, Digestion and Reproduction, Imperial College London, W2 1NY, London, United Kingdom; Department of Gastroenterology and Hepatology, Imperial College Healthcare NHS Trust, London, W2 1NY, United Kingdom; Department of Metabolism, Digestion and Reproduction, Imperial College London, W2 1NY, London, United Kingdom; Department of Metabolism, Digestion and Reproduction, Imperial College London, W2 1NY, London, United Kingdom; Department of Gastroenterology and Hepatology, Imperial College Healthcare NHS Trust, London, W2 1NY, United Kingdom; Department of Metabolism, Digestion and Reproduction, Imperial College London, W2 1NY, London, United Kingdom; Department of Gastroenterology and Hepatology, Imperial College Healthcare NHS Trust, London, W2 1NY, United Kingdom; Department of Metabolism, Digestion and Reproduction, Imperial College London, W2 1NY, London, United Kingdom; Department of Gastroenterology and Hepatology, Imperial College Healthcare NHS Trust, London, W2 1NY, United Kingdom; Department of Gastroenterology, London North West University Healthcare Trust, London, HA1 3UJ, United Kingdom; Department of Metabolism, Digestion and Reproduction, Imperial College London, W2 1NY, London, United Kingdom; Department of Gastroenterology and Hepatology, Imperial College Healthcare NHS Trust, London, W2 1NY, United Kingdom; Department of Metabolism, Digestion and Reproduction, Imperial College London, W2 1NY, London, United Kingdom; Department of Medicine, University of Cambridge, Cambridge, CB2 0AW, United Kingdom; Department of Gastroenterology, Cambridge University Hospitals NHS Trust, Cambridge, CB2 0QQ, United Kingdom; Department of Metabolism, Digestion and Reproduction, Imperial College London, W2 1NY, London, United Kingdom; Department of Gastroenterology and Hepatology, Imperial College Healthcare NHS Trust, London, W2 1NY, United Kingdom; Department of Metabolism, Digestion and Reproduction, Imperial College London, W2 1NY, London, United Kingdom; Department of Gastroenterology and Hepatology, Imperial College Healthcare NHS Trust, London, W2 1NY, United Kingdom; Department of Metabolism, Digestion and Reproduction, Imperial College London, W2 1NY, London, United Kingdom; Department of Gastroenterology and Hepatology, Imperial College Healthcare NHS Trust, London, W2 1NY, United Kingdom

**Keywords:** older adults, late-onset IBD, ulcerative colitis, Crohn’s disease

## Abstract

**Background:**

The incidence of inflammatory bowel disease (IBD) in older adults is rising. Studies of late-onset (LO) (diagnosis  ≥60 years of age) IBD are contradictory, with some suggesting increased use of corticosteroid and reduced use of advanced therapies. This multicenter study assessed disease phenotype and therapeutic choice in LO-IBD.

**Methods:**

Patients recruited to the NIHR IBD BioResource with LO ulcerative colitis (UC) or Crohn’s disease (CD) were compared with those with young-onset (YO) (16-39 years of age) and mid-onset (MO) (40-59 years of age) IBD. Medication use and surgeries were assessed in propensity score–matched cohorts.

**Results:**

A total of 32 012 patients were studied: 16 930 UC (2247 LO) and 15 082 CD (1409 LO). LO-UC was mostly left-sided, with less isolated proctitis. LO-CD patients had less ileocolonic and perianal disease. Corticosteroid, immunomodulator, and anti-tumor necrosis factor α (anti-TNF) use was lower in LO-UC and LO-CD; however, there was no difference in time to colectomy (LO-UC) or intestinal resection (LO-CD) compared with younger patients. LO-CD patients were more likely to receive vedolizumab and ustekinumab than YO-CD (odds ratio, 0.34 [95% confidence interval, 0.18-0.61] and 0.29 [95% confidence interval, 0.09-0.76], respectively). LO patients with perianal CD were less likely to receive anti-tumor necrosis factor or undergo perianal surgery than YO and MO patients with perianal CD (all *P < *.01).

**Conclusions:**

LO-IBD has a distinct phenotype, with less isolated proctitis (LO-UC) and less ileocolonic and perianal CD. LO-IBD patients had lower corticosteroid, immunomodulator, and anti-tumor necrosis factor use but equivalent colectomy and intestinal resection rates. LO-CD is notable for the higher use of vedolizumab and ustekinumab, suggesting that age, comorbidity, and disease phenotype affect biologic choice in older adults. Research to understand whether differential treatment of older adults is justified is crucial.

Key Messages *What is already known?*

Studies have reported a distinct phenotype of LO-IBD; however, data on use of therapies in these patients have been contradictory.

*What is new here?*

In LO-IBD patients, use of corticosteroids, immunomodulators, and anti-TNF was lower than younger patients.

LO-CD patients received more vedolizumab and ustekinumab.

*How can this study help patient care?*

These findings highlight a need to understand how disease phenotype may affect therapeutic outcomes in late-onset IBD, including research to determine whether differential treatment of LO-IBD is justified.

## Introduction

Life expectancy worldwide is increasing and is associated with rising prevalence of noncommunicable disease.^1^ The inflammatory bowel diseases (IBDs), ulcerative colitis (UC) and Crohn’s disease (CD), are chronic immune-mediated inflammatory conditions. IBD prevalence is rising, associated with factors including shifting sociocultural patterns to increasingly urban populations, and environmental exposures including dietary alterations and smoking.^2–5^ Although more commonly diagnosed in young adults, IBD incidence across age is bimodal, with around 10% to 15% of diagnoses seen in those older than 60 years of age,^6^^,^^7^ henceforth referred to as late-onset (LO) IBD. The older IBD population comprises those newly diagnosed, and those with younger-onset (YO) IBD surviving to older age, and is forecast to represent 25% to 30% of the total IBD population by 2030.^5^

LO-IBD management presents unique challenges, often impacted by comorbidities, polypharmacy, and the alterations in drug tolerability associated with aging.^6^^,^^7^ The designation of 60 years of age as the “cutoff” at which patients are considered “older” was endorsed in a 2017 European Crohn’s and Colitis Organization topical review, which also highlighted the importance of accounting for comorbidities rather than chronological age alone.^6^ However, the impact of these factors in older adults with IBD has not consistently been accounted for in registry studies to date, leading to potential bias.^8–11^

While phenotypic and treatment variations in LO-IBD have been reported, studies to date have been contradictory, and have not consistently accounted for the impact that phenotypic differences may have on treatment.^7^^,^^12–14^ In addition, older patients are underrepresented in clinical trials, leading to a paucity of data regarding treatment strategies in this cohort.^15^ Some studies have reported prolonged corticosteroid use in older adults with IBD, which are associated with increased mortality, and reduced use of biologics, with concerns around their safety in older adults.^7^^,^^8^^,^^14^^,^^16–20^ However, other studies have shown no differences in safety in selected older patients treated with biologics.^21^ The increased mortality associated with IBD-related hospitalization in older patients highlights the importance of optimal management of this complex cohort.^22^ More granular analysis of large cohorts is crucial to understand how disease phenotype may affect treatment in LO-IBD.

In this study, we analyzed LO-IBD in the largest European IBD-specific database, using the NIHR IBD BioResource (IBD-BR). The IBD phenotypes, treatment, and requirement for surgery were compared in LO and YO patients, identifying important phenotypic variations and a differential treatment of older adults with IBD.

## Methods

### Study population

This analysis used data from the IBD-BR, an open-access national platform prospectively recruiting patients across 118 UK centers.^23^ All patients in the IBD-BR (REC reference 15/EE/0286, IRAS 173561) provided signed consent. Documentation of the disease phenotype, medication history, comorbidity, and IBD-related surgery used a combination of case note review, patient interview, and patient-completed questionnaires, providing the most extensively characterized IBD database in the United Kingdom. At the time of data extraction (October 2025), 33 114 patients with IBD were included. Patients with confirmed UC or CD,  who were ≥16 years of age at diagnosis after January 1, 2000, were included, reflecting the biologic therapeutic era. Patients with IBD unclassified were excluded. The Cambridge Central Research Ethics Committee gave ethical approval for this study (ref. 15/EE/0268).

### Statistical analysis

All analyses were conducted in R Statistical Software (v4.4.2; R Foundation for Statistical Computing). Patients were grouped by age at diagnosis into those diagnosed between 16 and 39 years (YO-IBD), between 40 and 59 years of age (mid-onset [MO] IBD), and at ≥60 years of age (LO-IBD). The LO cohort was compared with groups of YO and MO patients. Descriptive statistics were used to summarize IBD phenotype (stratified by Montreal criteria) and demographic characteristics: sex, smoking history, body mass index (BMI) (BMI range: underweight, < 18.5 kg/m^2^; normal weight, 18.5-24.9 kg/m^2^; overweight, 25-29.9 kg/m^2^; obese, ≥30 kg/m^2^), and presence of comorbidity (cancer, lymphoma, cardiovascular disease [myocardial infarction, coronary artery disease, congestive heart failure], cerebrovascular disease, asthma, chronic obstructive pulmonary disease, multiple sclerosis, diabetes mellitus, hypertension, hypercholesterolemia, osteoarthritis, and rheumatoid arthritis).^24^ Chi-square tests and the Kruskal-Wallis test were used to analyze categorical and continuous variables, respectively. For variables with ≥2 outcomes (eg, UC extent), a post hoc chi-square test was performed using the chisq.posthoc.test package (v0.1.2), adjusted for multiple comparisons using Benjamini-Hochberg correction. Sensitivity analyses were conducted to examine the impact of longer disease duration on disease phenotype in patients with no significant difference in length of follow-up (8-13 years in CD, 9-15 years in UC). A 2-sided *P* value <.05 indicated statistical significance. Numbers in tables represent complete records for each variable.

### Propensity Score Matching

Medication use and surgery (see the following) were assessed in patients with similar characteristics by comparing propensity score–matched cohorts, using the MatchIt R package (v4.7.0).^25^ Propensity score matching (PSM) was based on a logistic regression including sex, smoking status, time since diagnosis (continuous variable), disease extent (UC), disease location and behavior (CD), and presence of perianal disease (CD). PSM was used separately to match both the YO and MO groups to the LO group in those with UC and CD, and separately for patients with perianal CD. PSM was also performed for patients with complex fistulizing perianal CD (complex perianal fistulae or abscess). The standardized mean difference after matching was <0.1 for almost all variables. In cases in which the standardized mean difference was >0.1, a sensitivity analysis was undertaken with exclusion of the relevant affected variable category.

### Medication use

Using multivariable logistic regression (finalfit R package, v1.0.8), any documented use of IBD-related medications (aminosalicylates [5-aminosalicylic acid (5-ASA)], oral corticosteroids [budesonide, prednisolone], immunomodulators [azathioprine, 6-mercaptopurine, methotrexate], anti-tumor necrosis factor α [TNF] agents [infliximab and adalimumab in CD; infliximab, adalimumab, and golimumab in UC], anti-integrins [vedolizumab], and anti-interleukin-12/23 inhibitors [ustekinumab in CD]) was compared between PSM groups. These analyses were performed separately for PSM patients with UC, CD, and perianal CD. A sensitivity analysis was performed analyzing medication use in complex fistulizing perianal disease. Multivariable logistic regression models were adjusted for important IBD variables, namely sex, smoking status, presence of a comorbidity, presence of extraintestinal manifestations (EIMs), disease duration (as a continuous variable), disease location (CD) or extent (UC), disease behavior (CD), and presence of perianal disease (CD).

Proportionate use of IBD-related medications was also analyzed in the unmatched UC and CD cohorts at 2 years and 5 years postdiagnosis, and all time. A time-trend analysis of IBD-medication use in all patients with IBD was performed (Figure S1).

Use of biologic therapies was analyzed from the date of UK drug approval: 2000 for anti-TNF in CD (infliximab approved 1999), 2008 for anti-TNF in UC (infliximab approved 2008), 2015 for vedolizumab in UC and CD, and 2017 for ustekinumab in CD.^26–29^

### Surgery

Cox proportional hazards models (finalfit R package, v1.0.8) were used to compare the hazard ratio (HR) for colectomy (UC), intestinal resection (CD), and perianal surgery (perianal CD) between PSM age groups, adjusted for the same variables as in medication use previously, and in addition use of corticosteroids, immunomodulators, and biologics. In a sensitivity analysis, the HR for perianal surgery was also assessed in PSM patients with complex fistulizing perianal CD. Differences in time to colectomy (UC), intestinal resection (CD), and perianal surgery (perianal CD) were compared with Kaplan-Meier curves and log-rank tests in PSM cohorts.

## Results

In total, 32 012 patients met the inclusion criteria: 16 930 with UC and 15 082 with CD (Tables S1 and S2, Figure S2).

### Ulcerative colitis

#### Demographics and phenotype

Disease phenotype and demographic characteristics are shown in Table 1 and Table S1, respectively. A total of 13.3% (n = 2247) UC patients were diagnosed at ≥60 years of age. Patients with LO-UC were significantly more likely to be male (56.5%), ex-smokers (62.1%), and overweight (40.6%) or obese (24.4%) compared with those with YO-UC (*P* < .001 for all). The proportion of patients with comorbidities increased with age, with more LO-UC patients having a history of cancer (29.4%) and cardiovascular (19.6%) and cerebrovascular (5.0%) disease than both younger groups (*P* < .001 for all). The overall proportion of patients with EIMs was equivalent between groups (YO-UC, 13.6%; MO-UC, 15.3%; LO-UC, 14.7%). Ankylosing spondylitis and enteropathic arthritis were significantly more common in LO-UC than YO-UC (*P* = .047 and .003, respectively). Median follow-up was 11 years (interquartile range [IQR]: 8-16 years) for YO-UC, 12 years (IQR: 8-17 years) for MO-UC, and 10 years (IQR: 7-13 years) for LO-UC. History of prior IBD-related hospital admission was significantly lower in LO-UC (28.4%) than both YO-UC (47.2%) and MO-UC (33.3%) (both *P* < .001). This finding persisted following sensitivity analysis controlling for length of follow-up (Table S3).

**Table 1:** Phenotype of young-onset (16-39 years), mid-onset (40-59 years), and late-onset (≥60 years) UC and CD.

	Young onset (16-39 y)	Mid onset (40-59 y)	Late onset (≥60 y)	*P* 16-39 y vs ≥60 y	*P* 40-59 y vs ≥60 y
**UC**					
**Total**	9480 (56.0)	5203 (30.7)	2247 (13.3)		
**UC disease extent**				**<.001**	**<.001**
** E1 (proctitis)**	1446 (17.7)	893 (19.3)	292 (14.5)	**.003**	**<.001**
** E2 (left-sided)**	4079 (49.3)	2402 (51.8)	1091 (54.2)	**<.001**	.22
** E3 (extensive)**	2734 (33.0)	1342 (28.9)	630 (31.3)	.42	.22
**CD**					
** Total**	9921 (65.8)	3752 (24.9)	1409 (9.3)		
**CD disease location**				**<.001**	**.002**
** L1 (ileal)**	3564 (37.3)	1640 (45.1)	632 (46.4)	**<.001**	1
** L2 (colonic)**	2618 (27.4)	1132 (31.1)	471 (34.6)	**<.001**	.14
** L3 (ileocolonic)**	3271 (34.2)	819 (22.5)	242 (17.8)	**<.001**	**.007**
** L4 (upper gastrointestinal)**	109 (1.1)	45 (1.2)	18 (1.3)	.35	1
**CD disease behavior**				**<.001**	**.002**
** Inflammatory**	6227 (66.8)	2240 (69.9)	946 (71.7)	.056	1
** Stricturing**	1985 (21.3)	793 (22.5)	303 (23.0)	.12	1
** Penetrating**	1107 (11.9)	293 (8.3)	70 (5.3)	**<.001**	.14
**Perianal disease**	3123 (33.8)	751 (21.5)	199 (15.2)	**<.001**	**<.001**
** Tags/fissures/ulcers**	1139 (11.5)	297 (7.9)	80 (5.7)	**<.001**	**.007**
** Perianal abscess**	1341 (13.5)	284 (7.6)	68 (4.8)	**<.001**	**.001**
** Simple fistula**	1033 (10.4)	217 (5.8)	49 (3.5)	**<.001**	**.001**
** Complex fistula**	770 (7.8)	156 (4.2)	36 (2.6)	**<.001**	**.009**

Values are n (%).

Abbreviations: CD, Crohn’s disease; UC, ulcerative colitis.

Bold was used to highlight significant values <0.05

Fewer patients with LO-UC had isolated proctitis than both YO-UC and MO-UC (LO, 14.5%; YO, 17.7% [*P* = .003]; MO, 19.3% [*P* < .001]). LO-UC was most often left-sided (54.2%), and the proportion of patients with extensive disease was equivalent between all groups (YO-UC, 33.0%; MO-UC, 28.9%; LO-UC, 31.3%). In a sensitivity analysis assessing the impact of longer disease duration, left-sided UC remained predominant (55%), with no difference in extensive disease compared with both younger groups (Table S4).

#### Medications and surgery

Results for use of medications in matched groups are shown in Table 2, and proportionate use in the whole unmatched cohort in Table S5. There was no difference in use of 5-ASA in LO-UC compared with YO and MO-UC (*P* = .87 and .23, respectively). There was lower use of corticosteroids, immunomodulators, anti-TNF, and anti-TNF/immunomodulator combination therapy in LO-UC compared with both younger cohorts (*P* values between <.001 and .009). There was no significant difference in use of vedolizumab in LO-UC compared with both YO-UC and MO-UC (*P* = .92 and .55, respectively).

**Table 2:** Adjusted or for medication use in propensity score–matched patients with young-onset (16-39 years), mid-onset (40-59 years), and late-onset (≥60 years) ulcerative colitis.

	Age group	No	Yes	Multivariable OR (95% CI)	*P*
**5-ASA**	Young onset	199 (14.7)	1157 (85.3)	1.02 (0.80-1.30)	.87
	Mid onset	224 (17.1)	1086 (82.9)	0.87 (0.70-1.09)	.23
	Late onset	269 (16.1)	1406 (83.9)	—	—
**Corticosteroids**	Young onset	472 (34.8)	884 (65.2)	1.67 (1.40-1.99)	**<.001**
	Mid onset	542 (41.4)	768 (58.6)	1.24 (1.06-1.47)	**.009**
	Late onset	776 (46.3)	899 (53.7)	—	—
**Immunomodulator**	Young onset	652 (48.1)	704 (51.9)	2.83 (2.36-3.39)	**<.001**
	Mid onset	743 (56.7)	567 (43.3)	1.73 (1.46-2.06)	**<.001**
	Late onset	947 (68.9)	427 (31.1)	—	—
**Anti-TNF**	Young onset	750 (61.7)	465 (38.3)	3.60 (2.89-4.49)	**<.001**
	Mid onset	843 (72.3)	323 (27.7)	1.99 (1.61-2.47)	**<.001**
	Late onset	1253 (82.7)	263 (17.3)	—	—
**Anti-TNF and immunomodulator**	Young onset	870 (74.7)	294 (25.3)	4.22 (3.24-5.54)	**<.001**
	Mid onset	920 (80.6)	221 (19.4)	2.68 (2.07-3.48)	**<.001**
	Late onset	1337 (90.7)	137 (9.3)	—	—
**Vedolizumab**	Young onset	588 (85.3)	101 (14.7)	1.02 (0.70-1.48)	.92
	Mid onset	568 (87.1)	84 (12.9)	0.90 (0.62-1.28)	.55
	Late onset	715 (81.8)	159 (18.2)	—	—

ORs report risk of treatment use in young-onset and mid-onset ulcerative colitis relative to late-onset ulcerative colitis.

Abbreviations: CI, confidence interval; OR, odds ratio; TNF; tumor necrosis factor; 5-ASA, 5-aminosalicylic acid.

There was no difference in time to colectomy in LO-UC compared with both younger cohorts (log-rank *P* = .60) (Figure 1A; Table S6) and no overall difference in the HR for colectomy for LO-UC compared with YO-UC (HR, 1.22 [95% confidence interval [CI], 0.76-1.96]; *P* = .42) or MO-UC (HR, 1.10 [95% CI, 0.69-1.74]; *P* = .68) (Table 3). Acute severe UC was the most common indication for colectomy in all age groups (YO, 53.1%; MO, 47.0%; LO, 52.3%) (both *P* = 1). LO-UC patients were significantly more likely to undergo colectomy for colorectal cancer than YO-UC patients (LO, 8.0%; YO, 1.5%) (*P* = .045) and less likely to undergo colectomy for chronic continuous colitis than YO-UC (LO, 12.5%; YO, 26.2%) (*P = *.045) (Table 4). These findings persisted following sensitivity analysis controlling for disease duration (Table S7).

**Figure 1:**
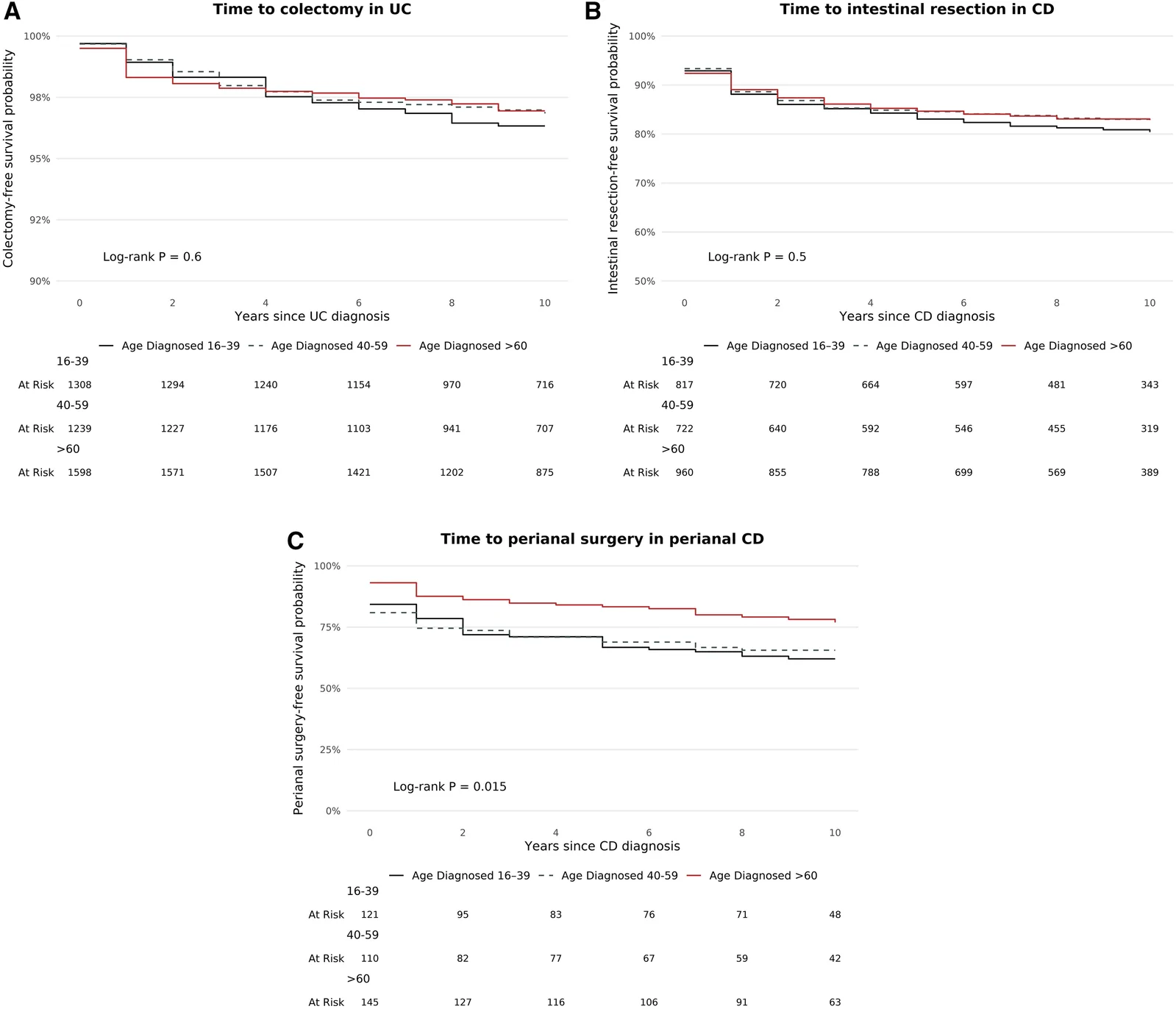
Kaplan-Meier curves showing time to surgery in propensity score–matched groups. (A) Colectomy in young-onset (YO) (16-39 years of age), mid-onset (MO) (40-59 years of age), and late-onset (LO) (60 years of age) ulcerative colitis (UC). (B) Intestinal resection in YO Crohn’s disease (CD), MO-CD, and LO-CD. (C) Perianal surgery in YO, MO, and LO perianal CD.

**Table 3:** Cox proportional hazards regression analysis between propensity score–matched cohorts, comparing risk of colectomy (UC), intestinal resection (CD), and perianal surgery (perianal CD).

		n (%)	Multivariable HR (95% CI)	*P*
**Colectomy (UC)**	Young onset	1308 (31.6)	1.22 (0.76-1.96)	.42
	Mid onset	1239 (29.9)	1.10 (0.69-1.74)	.68
	Late onset	1598 (38.6)	—	—
**Intestinal resection (CD)**	Young onset	817 (32.7)	1.15 (0.90-1.47)	.25
	Mid onset	722 (28.9)	0.91 (0.72-1.16)	.46
	Late onset	1823 (38.4)	—	—
**Perianal surgery (perianal CD)**	Young onset	136 (32.5)	2.09 (1.29-3.39)	**.003**
	Mid onset	126 (30.1)	2.06 (1.31-3.25)	**.002**
	Late onset	156 (37.3)	—	—

The n (%) refers to the number and proportion of propensity-matched patients of each age group following propensity score matching that were included in the regression models. Adjusted HRs are shown for young-onset and mid-onset groups, relative to late onset.

Abbreviations: CD, Crohn’s disease; CI, confidence interval; HR, hazard ratio; UC, ulcerative colitis.

**Table 4:** Indication for colectomy in young-onset, mid-onset, and late-onset UC.

	Young onset (16-39 y)	Mid onset (40-59 y)	Late onset (≥60 y)	*P* 16-39 y vs ≥60 y	*P* 40-59 y vs ≥60 y
**Colectomy indication**				**.003**	.099
** Acute severe UC**	254 (53.1)	93 (47.0)	46 (52.3)	1	1
** Chronic continuous colitis**	125 (26.2)	48 (24.2)	11 (12.5)	**.045**	.21
** Colorectal cancer**	7 (1.5)	9 (4.5)	7 (8.0)	**.045**	.50

Values are n (%).

Abbreviation: UC, ulcerative colitis.

### Crohn’s disease

#### Demographics and phenotype

CD phenotype and patient characteristics are shown in Table 1 and Table S2, respectively. The LO group comprised 9.3% (n = 1409) of CD patients diagnosed at ≥60 years of age. CD patients were more often female in all age groups (YO, 53.4%; MO, 56.2%; LO, 53.4%). LO-CD patients were less likely to be active smokers (12.6%) than both YO-CD (15.4%; *P = *.022) and MO-CD (18.2%; *P* < .001) patients, and more likely to be overweight than those with YO-CD (LO, 36.9%; YO, 29.3%) (*P* < .001). In LO-CD, 58.0% had comorbidities compared with 23.4% with YO-UC and 39.8% with MO-CD (both *P* < .001). EIMs were present in 20.4% of LO-CD patients, compared with 21.9% with YO-CD (*P = *.30) and 24.3% with MO-CD (*P = *.011). Median length of follow-up was 12 years (IQR: 8-18 years) for YO-CD, 11 years (IQR: 7-16 years) for MO-CD, and 9 years (IQR: 6-12 years) for LO-CD (both *P* < .001). A history of prior IBD-related hospital admission was significantly lower in LO-CD (38.8%) than in both YO-CD (58.9%) and MO-CD (45.5%; both *P* < .001).

Patients with LO-CD most often had ileal (46.4%) and colonic (34.6%) disease, with less ileocolonic (17.8%) and perianal disease (15.2%) than those with YO-CD (34.2% with ileocolonic disease and 33.8% with perianal disease; both *P* < .001) and MO-CD (22.5% with ileocolonic disease; *P = *.007; 21.5% with perianal disease; *P* < .001). Perianal lesions of all subtypes (tags/fissures/ulcers, perianal abscess, simple and complex fistulae) were significantly less common in LO-CD patients compared with both YO-CD and MO-CD patients. CD behavior was predominantly inflammatory in all groups, with less penetrating disease in LO-CD than YO-CD (YO, 11.9%; LO, 5.3%) (*P* < .001). In sensitivity analyses assessing the impact of longer disease duration on CD phenotype (Tables S4 and S8), LO-CD disease location remained distinct from YO-CD; there was more stricturing (LO 22% vs YO 18.5%; *P* < .001) and less penetrating behavior (LO 5.3% vs YO 11.2%; *P* < .001).

#### Medications and surgery

Results for use of medications in matched groups with CD and perianal CD are shown in Table 5, and proportionate medication use for the whole unmatched cohort is shown in Table S5. In LO-CD, there was no significant difference in 5-ASA use between groups. There was less use of corticosteroid in LO-CD than YO-CD (odds ratio [OR] 1.65 [95% CI, 1.32-2.07], *P* < .001), but no significant difference in LO-CD and MO-CD (OR, 1.21 [95% CI, 0.97-1.50]; *P* = .084). Immunomodulator, anti-TNF, and anti-TNF/immunomodulatory combination therapy was lower in LO-CD compared with both YO and MO-CD (*P* < .001 for all). Vedolizumab use was more common in LO-CD (YO vs LO: OR, 0.34 [95% CI, 0.18-0.61]; *P* < .001; MO vs LO: OR, 0.46 [95% CI, 0.25-0.80]; *P* = .007). Ustekinumab use was higher in LO-CD compared with YO-CD (OR, 0.29 [95% CI, 0.09-0.76]; *P* = .018) but not MO-CD (OR, 0.61 [95% CI, 0.25-1.40]; *P* = .26).

**Table 5:** Adjusted OR for medication use in propensity score–matched patients with young-onset (16-39 years), mid-onset (40-59 years), and late-onset (≥60 years) Crohn’s disease and perianal Crohn’s disease.

	Age group	No	Yes n (%)	Multivariable (95% CI)	*P*
**Crohn’s disease**					
**5-ASA**	Young onset	569 (66.6)	285 (33.4)	0.89 (0.70-1.12)	.32
	Mid onset	501 (66.4)	253 (33.6)	0.87 (0.69-1.10)	.24
	Late onset	495 (61.2)	314 (38.8)	—	—
**Corticosteroids**	Young onset	274 (32.1)	580 (67.9)	1.65 (1.32-2.07)	**<.001**
	Mid onset	278 (36.9)	476 (63.1)	1.21 (0.97-1.50)	.084
	Late onset	416 (41.5)	586 (58.5)	—	—
**Immunomodulator**	Young onset	241 (28.2)	613 (71.8)	2.55 (2.02-3.23)	**<.001**
	Mid onset	244 (32.4)	510 (67.6)	1.88 (1.50-2.36)	**<.001**
	Late onset	497 (49.6)	505 (50.4)	—	—
**Anti-TNF**	Young onset	389 (45.6)	465 (54.4)	2.70 (2.15-3.40)	**<.001**
	Mid onset	383 (50.8)	371 (49.2)	2.07 (1.65-2.59)	**<.001**
	Late onset	660 (65.9)	342 (34.1)	—	—
**Anti-TNF and immunomodulator**	Young onset	466 (58.4)	332 (41.6)	3.40 (2.63-4.40)	**<.001**
	Mid onset	466 (65.7)	243 (34.3)	2.34 (1.82-3.03)	**<.001**
	Late onset	771 (81.1)	180 (18.9)	—	—
**Vedolizumab**	Young onset	439 (94.6)	25 (5.4)	0.34 (0.18-0.61)	**<.001**
	Mid onset	368 (92.5)	30 (7.5)	0.46 (0.25-0.80)	**.007**
	Late onset	474 (83.9)	91 (16.1)	—	—
**Ustekinumab**	Young onset	272 (95.1)	14 (4.9)	0.29 (0.09-0.76)	**.018**
	Mid onset	222 (94.1)	14 (5.9)	0.61 (0.25-1.40)	.26
	Late onset	313 (89.9)	35 (10.1)	—	—
**Perianal Crohn’s disease**					
**5-ASA**	Young onset	102 (75.0)	34 (25.0)	0.50 (0.27-0.93)	**0.029**
	Mid onset	84 (66.7)	42 (33.3)	0.80 (0.44-1.43)	.45
	Late onset	95 (60.9)	61 (39.1)	—	—
**Corticosteroids**	Young onset	55 (40.4)	81 (59.6)	1.49 (0.85-2.61)	.17
	Mid onset	51 (40.5)	75 (59.5)	1.21 (0.70-2.08)	.49
	Late onset	75 (48.1)	81 (51.9)	—	—
**Immunomodulator**	Young-onset	21 (15.4)	115 (84.6)	4.01 (2.01-8.32)	**<.001**
	Mid-onset	37 (29.4)	89 (70.6)	1.32 (0.73-2.41)	.36
	Late-onset	63 (40.4)	93 (59.6)	—	—
**Anti-TNF**	Young onset	32 (23.5)	104 (76.5)	2.97 (1.61-5.63)	**.001**
	Mid onset	32 (25.4)	94 (74.6)	2.40 (1.32-4.44)	**.005**
	Late onset	28 (37.8)	46 (62.2)	—	—
**Anti-TNF and immunomodulator**	Young onset	47 (37.3)	79 (62.7)	4.59 (2.50-8.66)	**<.001**
	Mid onset	58 (50.0)	58 (50.0)	2.28 (1.27-4.18)	**.006**
	Late onset	96 (66.7)	48 (33.3)	—	—

Values are n (%). ORs report risk of treatment use in young-onset and mid-onset patients, relative to late onset. The n (%) refers to the number and proportion of patients of each age group following propensity score–matching that were included in the regression models. Adjusted ORs are shown for young-onset and mid-onset groups, relative to late onset.

Abbreviations: CI, confidence interval; OR, odds ratio; TNF; tumor necrosis factor; 5-ASA, 5-aminosalicylic acid.

In PSM patients with perianal CD, corticosteroid use was equivalent between groups. Anti-TNF and anti-TNF/immunomodulator combination therapy were significantly reduced in LO-CD compared with both younger groups. LO-CD patients with complex fistulizing perianal disease were as likely to receive anti-TNF as MO-CD patients (OR, 1.88 [95% CI, 0.80-4.53]; *P* = .15) (Table S9).

There was no difference in time to intestinal resection in LO-CD compared with both younger groups (log-rank *P = *.50) (Figure 1B) and no difference in the HR for intestinal resection between groups (YO vs LO: HR, 1.15 [95% CI, 0.90-1.47]; *P* = .25; MO vs LO: HR, 0.91 [95% CI, 0.72-1.16]; *P* = .46) (Table 3). In perianal CD, the HR for perianal surgery was lower in LO-CD compared with both YO-CD (HR, 2.09 [95% CI, 1.29-3.39]; *P = *.003) and MO-CD (HR, 2.06 [95% CI, 1.31-3.25]; *P* = .002) (Table 3), and there was a longer time to perianal surgery in LO-CD than YO-CD (LO vs YO, log-rank *P* = .005) (Figure 1C;  Table S6). A lower HR for perianal surgery in LO-CD was also found in sensitivity analyses including only patients with complex fistulizing perianal disease (Table S10).

## Discussion

Given the rising burden of IBD and increasingly older populations worldwide, understanding the phenotypic features of LO-IBD is crucial.^1–3^ Although incidence of LO-UC may be declining in some Western societies, incidence of LO-CD and the overall prevalence of IBD in older adults continues to rise.^30^ This study found distinct phenotypic variations in LO-IBD and highlights important differences in treatment between LO and younger onset patients with similar disease phenotypes.

Specific phenotypic patterns of LO-IBD have been reported.^12^ In a meta-analysis, Ananthakrishnan et al^12^ found a slightly increased frequency of left-sided UC and equal proportions of isolated proctitis and extensive disease compared with YO-UC. They also reported less ileocolonic and perianal disease, and less penetrating behavior, in LO-CD.^12^ A large Swedish registry study contradicted these findings, documenting less isolated proctitis and extensive disease in LO-UC and similar disease location in LO-CD; however, 28% of the elderly UC patients included had undefined disease location.^14^

In this study, which compared 3 age cohorts, we found more pronounced differences between LO and YO patients. In both UC and CD, there were fewer never smokers and more overweight individuals in the LO cohorts. Disease phenotype differed across groups: fewer LO-UC patients had isolated proctitis compared with both YO-UC and MO-UC patients. LO-CD patients had more ileal and colonic, and less ileocolonic, perianal and penetrating disease than YO-CD patients. Perianal lesions of all subtypes were less common than both younger groups. That this difference persisted after controlling for longer length of follow-up, which additionally highlighted an increased stricturing phenotype in LO-CD, suggests these are unique characteristics distinguishing YO-CD and LO-CD.

Existing studies on the treatment of older adults with IBD have also been contradictory. With the proliferation of biologic therapies over the last 2 decades, limiting the use of corticosteroids, with their multiple side effects, has become a focus of management. Despite this, some studies have shown increased corticosteroid use, with repeated or prolonged courses in older adults,^14^^,^^31^^,^^32^ which are associated with a higher risk of infection and mortality.^17^ A meta-analysis, however, showed comparable corticosteroid use in older patients with IBD at 5 years.^13^ We have demonstrated significantly less corticosteroid use in LO-UC than both younger cohorts, and less use in LO-CD than YO-CD. Notably, Everhov et al^14^ analyzed data from 2006 to 2013, so greater awareness of the risks of corticosteroid use and increased availability of biologics over the last decade may have contributed to the reduction seen.

Use of biologic and immunosuppressant therapies in older populations has historically been low, with immunosuppressants associated with well documented risks of infection, lymphoma, and nonmelanoma skin cancer.^11^^,^^13^^,^^14^^,^^33^^,^^34^ However, the evidence base supporting use of biologics in older patients has grown, and despite an increased risk of infection, there is no increase in risk of malignancy or mortality.^35^ In this study, thiopurine and anti-TNF use were lower in LO-IBD compared with the younger patients; however, LO-CD patients were more likely to receive vedolizumab and ustekinumab than YO-CD patients. Increased use of vedolizumab is not surprising, with its supposed lower systemic immunosuppressive effects, according to its mechanism of action, offering an attractive prospect for older adults.^36^^,^^37^ Ustekinumab has also been shown to have comparable efficacy and safety compared with anti-TNF and vedolizumab in small studies of older CD patients.^38^^,^^39^ However, whether increased use of vedolizumab in LO-CD is justified requires further investigation, particularly given the absence of any reduction in serious infections compared with anti-TNF in a study by Kirchgesner et al.^40^ In this study, use of anti-TNF in older adults was influenced by disease phenotype, with equivalent use in LO and MO patients with complex fistulizing CD, suggesting that physicians’ willingness to use anti-TNF in older patients may be influenced by consideration of a higher benefit vs risk.

Requirement for surgery in LO-IBD is debated, with both increased^10^^,^^11^^,^^14^ and equal^7^ rates reported. A meta-analysis showed equivalent 5-year colectomy rates in LO-UC, and 5-year surgery rates in LO-CD, compared with younger patients.^13^ A different study reported higher colectomy rates in UC.^12^ The majority of studies included had not accounted for disease extent and behavior, which can influence risk of surgery.^7^ In this study, there was no difference in colectomy and intestinal resection rates in LO-UC and CD. Perianal surgery was significantly lower in LO-CD compared with both younger groups, a finding similar to that of Ben Hur et al.^11^ We also found reduced use of perianal surgery in LO patients with complex fistulizing perianal disease. Ben Hur et al hypothesized that the observed reduction could relate to lower levels of perianal disease in the LO patients but lacked phenotypic data to support this view. Our study has demonstrated less perianal disease in LO-CD, and in these patients the rate of perianal surgery was significantly reduced compared with the younger cohorts. Whether this reduction relates to a lower requirement for surgery or physicians’/patients’ reluctance to employ perianal surgery requires further investigation.

This is the largest European study to assess treatment of LO-IBD in patients accounting for demographic factors, comorbidity, and disease phenotype. Strengths of this study are the extensively documented phenotypic and treatment data of patients managed in both secondary and tertiary care settings throughout the United Kingdom. Treatment decisions were unaffected by participation in the study and capture the combined practice of IBD clinicians nationally. The management was compared for around a decade post-diagnosis, and with PSM, demographic, and phenotypic features that may affect treatment decisions were accounted for. We acknowledge the limitations of this study, including the potential for selection bias in patient recruitment, such as patients with mobility or cognitive impairment, which may affect the representation of more highly comorbid patients within the study. We also acknowledge that we have not been able to use validated frailty scores to assess its impact on treatment, and although extensive medication data were assessed, we were not able to account for prescriptions made in primary care. However, these data constitute one of the largest and best characterized studies of LO-IBD to date, highlighting important findings and avenues for further research. These include studies to assess whether time to diagnosis in older patients differs from young patients, which could impact on phenotype and response to treatments. There is also an unmet need for analysis of large cohorts to better characterize any differences in medication efficacy and safety in LO-IBD, and inform on the positioning of advanced therapies.

We found a unique phenotype of LO-IBD, which was most distinct from YO-IBD. LO-UC was predominantly left-sided, with less isolated proctitis. LO-CD was characterized by less ileocolonic, perianal, and penetrating disease. Corticosteroid, immunomodulator, and anti-TNF use was lower in LO-IBD compared with younger patients. Increased vedolizumab and ustekinumab use in LO-CD suggests that a combination of age, comorbidity, and disease phenotype affect biologic treatment choice in older adults. Further studies to understand whether differences in biologic efficacy and adverse events justify this differential treatment of LO-IBD are essential.

## Supplementary Material

izag072_Supplementary_Data

## Data Availability

Patient-level data underlying this study are available to researchers subject to the access processes of the NIHR IBD BioResource, detailed further at https://www.ibdbioresource.nihr.ac.uk/index.php/resources.
