# Supplementary material for: Disease phenotype affects treatment of late-onset inflammatory bowel disease: Analysis of a large UK cohort
Source: Inflamm Bowel Dis. 2026 May 8;32(10):1888–97. doi: 10.1093/ibd/izag072 (PMC13626234; doi:10.1093/ibd/izag072)
Supplement: Supplementary file 1 [file izag072_supplementary_data.zip › Supplementary_materials_V2.0.docx]

Disease phenotype affects treatment of late-onset inflammatory bowel disease:

Analysis of a large UK cohort

**Supplementary Materials**

**Supplementary Table 1. Demographic characteristics in young-onset (16-39 years), mid-onset (40-59 years) and late-onset (≥60 years) ulcerative colitis.**

|  | Young-onset  16-39 years | Mid-onset  40-59 years | Late-onset  ≥60 years | *P*  (16-39 vs ≥60) | *P*  (40-59 vs ≥60) |
| --- | --- | --- | --- | --- | --- |
| Total, n (%) | 9480 (56.0) | 5203 (30.7) | 2247 (13.3) |  |  |
| Sex, n (%) |  |  |  | **<0.001** | **0.044** |
| Male | 3815 (48.6) | 2337 (53.7) | 1079 (56.5) |  |  |
| Female | 4042 (51.4) | 2017 (46.3) | 832 (43.5) |  |  |
| Age at diagnosis, Median (IQR) | 27.0 (22.0 to 33.0) | 48.0 (44.0 to 54.0) | 66.0 (62.0 to 70.0) | **<0.001** | **<0.001** |
| Years since diagnosis, Median (IQR) | 11.0 (8.0 to 16.0) | 12.0 (8.0 to 17.0) | 10.0 (7.0 to 13.0) | **<0.001** | **<0.001** |
| Years since diagnosis, years |  |  |  | **<0.001** | **<0.001** |
| <5 | 1008 (11.1) | 475 (9.5) | 296 (13.6) | **0.004** | **<0.001** |
| 5-10 | 3261 (35.9) | 1732 (34.6) | 982 (45.1)) | **<0.001** | **<0.001** |
| 10-15 | 2474 (27.2) | 1414 (28.3) | 642 (29.5) | 0.095 | 0.89 |
| 15-20 | 1240 (13.6) | 756 (15.1) | 186 (8.5) | **<0.001** | **<0.001** |
| >20 | 1113 (12.2) | 623 (12.5) | 71 (3.3) | **<0.001** | **<0.001** |
| Smoking status, n (%) |  |  |  | **<0.001** | **<0.001** |
| Never smoker | 5642 (62.1) | 2032 (40.4) | 758 (34.9) | **<0.001** | **<0.001** |
| Current smoker | 542 (6.0) | 241 (4.8) | 64 (2.9) | **<0.001** | **<0.001** |
| Former smoker | 2895 (31.9) | 2758 (54.8) | 1349 (62.1) | **<0.001** | **<0.001** |
| BMI, Median (IQR) | 24.9 (22.2 to 28.4) | 27.1 (24.3 to 30.7) | 26.6 (23.9 to 29.8) | **<0.001** | **<0.001** |
| BMI range kg/m^2^, n (%) |  |  |  | **<0.001** | **0.011** |
| <18.5 | 104 (2.4) | 29 (2.4) | 21 (1.6) | 0.34 | 0.38 |
| 18.5-24.9 | 2112 (48.3) | 845 (29.8) | 428 (33.4) | **<0.001** | 0.12 |
| 25-29.9 | 1366 (31.3) | 1124 (39.6) | 520 (40.6) | **<0.001** | 1.0 |
| >30 | 788 (18.0) | 841 (29.6) | 313 (24.4) | **<0.001** | **0.007** |
| IBD-related hospital admission | 4213 (47.2) | 1639 (33.3) | 603 (28.4) | **<0.001** | **<0.001** |
| Any comorbidity, n (%) | 2075 (21.9) | 2129 (40.9) | 1326 (59.0) | **<0.001** | **<0.001** |
| Hypertension | 213 (4.0) | 802 (23.3) | 678 (40.8) | **<0.001** | **<0.001** |
| Hypercholesterolaemia | 137 (2.6) | 683 (19.9) | 493 (29.7) | **<0.001** | **<0.001** |
| Diabetes | 95 (1.8) | 342 (9.9) | 269 (16.2) | **<0.001** | **<0.001** |
| Asthma | 864 (16.2) | 523 (15.2) | 255 (15.3) | 0.40 | 0.91 |
| COPD | 3 (0.1) | 21 (0.7) | 46 (3.1) | **<0.001** | **<0.001** |
| Cerebrovascular disease | 21 (0.4) | 77 (2.2) | 83 (5.0) | **<0.001** | **<0.001** |
| Cardiovascular disease | 53 (1.0) | 229 (6.6) | 326 (19.6) | **<0.001** | **<0.001** |
| Coronary artery disease | 42 (0.8) | 181 (5.2) | 269 (16.2) | **<0.001** | **<0.001** |
| Myocardial infarction | 7 (0.1) | 63 (1.8) | 84 (5.1) | **<0.001** | **<0.001** |
| Congestive heart failure | 5 (0.1) | 12 (0.3) | 21 (1.3) | **<0.001** | **<0.001** |
| Multiple sclerosis | 14 (0.2) | 16 (0.4) | 4 (0.2) | 1 | 0.44 |
| Arthritis | 250 (4.7) | 507 (14.7) | 347 (20.9) | **<0.001** | **<0.001** |
| Rheumatoid Arthritis | 127 (2.4) | 207 (6.0) | 119 (7.2) | **<0.001** | 0.13 |
| Lymphoma | 10 (0.1) | 19 (0.5) | 15 (0.8) | **<0.001** | 0.12 |
| Cancer | 905 (17.0) | 710 (20.6) | 488 (29.4) | **<0.001** | **<0.001** |
| Any EIM, n (%) | 970 (13.6) | 620 (15.3) | 257 (14.7) | 0.28 | 0.57 |
| Ankylosing spondylitis | 103 (1.4) | 93 (2.3) | 38 (2.1) | **0.047** | 0.82 |
| Enteropathic arthritis | 288 (4.0) | 225 (5.5) | 101 (5.7) | **0.003** | 0.83 |
| Psoriasis | 319 (4.4) | 211 (5.1) | 88 (4.9) | 0.44 | 0.79 |
| Erythema nodosum | 71 (1.0) | 26 (0.6) | 11 (0.6) | 0.17 | 1 |
| Iritis | 108 (1.5) | 118 (2.9) | 34 (1.9) | 0.29 | **0.037** |
| Orofacial granulomatosis | 28 (0.4) | 13 (0.3) | 1 (0.1) | **0.045** | 0.11 |
| Primary sclerosing cholangitis | 192 (2.7) | 50 (1.2) | 20 (1.1) | **<0.001** | 0.85 |

**Supplementary Table 2. Demographic characteristics in young-onset (16-39 years), mid-onset (40-59 years) and late-onset (≥ 60 years) Crohn’s disease.**

|  | Young-onset  16-39 years | Mid-onset  40-59 years | Late-onset  ≥60 years | *P*  (16-40 vs ≥60) | *P*  (40-59 vs ≥60) |
| --- | --- | --- | --- | --- | --- |
| Total, n (%) | 9921 (65.8) | 3752 (24.9) | 1409 (9.3) |  |  |
| Sex, n (%) |  |  |  | 1.0 | 0.10 |
| Male | 3879 (46.6) | 1136 (43.8) | 544 (46.6) |  |  |
| Female | 4438 (53.4) | 1767 (56.2) | 623 (53.4) |  |  |
| Age at diagnosis, Median (IQR) | 25.0 (20.0 to 31.0) | 48.0 (44.0 to 53.0) | 66.0 (63.0 to 71.0) | **<0.001** | **<0.001** |
| Years since diagnosis, median (IQR) | 12.0 (8.0 to 18.0) | 11.0 (7.0 to 16.0) | 9.0 (6.0 to 12.0) | **<0.001** | **<0.001** |
| Years since diagnosis, grouped |  |  |  | **<0.001** | **<0.001** |
| <5 | 940 (9.9) | 429 (11.9) | 227 (16.5) | **<0.001** | **<0.001** |
| 5-10 | 2981 (31.4) | 1322 (36.5) | 641 (46.7) | **<0.001** | **<0.001** |
| 10-15 | 2528 (26.6) | 994 (27.5) | 391 (28.5) | 0.43 | 1 |
| 15-20 | 1481 (15.6) | 490 (11.9) | 83 (6.0) | **<0.001** | **<0.001** |
| >20 | 1577 (16.6) | 383 (10.6) | 32 (2.3) | **<0.001** | **<0.001** |
| Smoking status, n (%) |  |  |  | **<0.001** | **<0.001** |
| Never smoker | 5229 (54.6) | 1359 (37.2) | 472 (34.4) | **<0.001** | 0.16 |
| Current smoker | 1474 (15.4) | 662 (18.2) | 173 (12.6) | **0.022** | **<0.001** |
| Former smoker | 2879 (30.0) | 1619 (44.5) | 727 (53.0) | **<0.001** | **<0.001** |
| BMI, median (IQR) | 25.1 (22.2 to 29.1) | 27.2 (23.9 to 31.2) | 26.2 (23.1 to 29.7) | **<0.001** | **<0.001** |
| BMI range kg/m^2^, n (%) |  |  |  | **<0.001** | **0.002** |
| <18.5 | 157 (3.5) | 44 (2.2) | 19 (2.5) | 0.60 | 1 |
| 18.5-24.9 | 2085 (46.2) | 646 (32.5) | 279 (37.2) | **<0.001** | 0.13 |
| 25-29.9 | 1324 (29.3) | 679 (34.2) | 277 (36.9) | **<0.001** | 0.48 |
| >30 | 950 (21.0) | 617 (31.1) | 175 (23.3) | 0.60 | **0.001** |
| IBD-related hospital admission | 5435 (58.9) | 1606 (45.5) | 507 (38.8) | **<0.001** | **<0.001** |
| Any comorbidity, n (%) | 2318 (23.4) | 1492 (39.8) | 817 (58.0) | **<0.001** | **<0.001** |
| Hypertension | 227 (2.3) | 547 (14.6) | 408 (29.1) | **<0.001** | **<0.001** |
| Hypercholesterolaemia | 116 (1.2) | 357 (9.5) | 274 (19.6) | **<0.001** | **<0.001** |
| Diabetes | 90 (1.6) | 216 (8.9) | 150 (15.0) | **<0.001** | **<0.001** |
| Asthma | 1041 (19.0) | 406 (16.8) | 154 (15.4) | **0.008** | 0.33 |
| COPD | 5 (0.1) | 39 (1.9) | 30 (3.4) | **<0.001** | **0.02** |
| Cerebrovascular disease | 22 (0.4) | 48 (2.0) | 56 (5.6) | **<0.001** | **<0.001** |
| Cardiovascular disease | 58 (1.1) | 137 (5.7) | 187 (18.8) | **<0.001** | **<0.001** |
| Coronary artery disease | 42 (0.8) | 101 (4.2) | 140 (14.0) | **<0.001** | **<0.001** |
| Myocardial infarction | 8 (0.1) | 35 (1.4) | 52 (5.2) | **<0.001** | **<0.001** |
| Congestive heart failure | 5 (0.1) | 10 (0.4) | 12 (1.2) | **<0.001** | **0.018** |
| Multiple sclerosis | 16 (0.2) | 13 (0.4) | 8 (0.7) | **0.005** | 0.37 |
| Arthritis | 409 (7.4) | 413 (17.1) | 273 (27.2) | **<0.001** | **<0.001** |
| Rheumatoid Arthritis | 203 (3.7) | 173 (7.2) | 83 (8.3) | **<0.001** | 0.28 |
| Lymphoma | 14 (0.2) | 19 (0.7) | 13 (1.2) | **<0.001** | 0.13 |
| Cancer | 976 (17.8) | 520 (21.5) | 326 (32.6) | **<0.001** | **<0.001** |
| Any EIM, n (%) | 1682 (21.9) | 702 (24.3) | 218 (20.4) | 0.30 | **0.011** |
| Ankylosing spondylitis | 209 (2.7) | 136 (4.7) | 49 (4.6) | **0.001** | 0.94 |
| Enteropathic arthritis | 571 (7.4) | 261 (9.0) | 69 (6.5) | 0.28 | **0.011** |
| Psoriasis | 536 (6.9) | 223 (7.4) | 84 (7.8) | 0.36 | 0.90 |
| Erythema nodosum | 276 (3.6) | 55 (1.9) | 11 (1.0) | **<0.001** | 0.94 |
| Iritis | 266 (3.5) | 121 (4.2) | 35 (3.2) | 0.80 | 0.21 |
| Orofacial granulomatosis | 222 (2.9) | 61 (2.1) | 16 (1.5) | 0.01 | 0.26 |
| Primary sclerosing cholangitis | 58 (0.8) | 23 (0.8) | 10 (0.9) | 0.67 | 0.82 |

**Supplementary Table 3. Sensitivity analysis showing demographic characteristics in young-onset (16-39 years), mid-onset (40-59 years) and late-onset (≥60 years) ulcerative colitis with no difference in disease duration (between 9-15 years in all groups).**

|  | Young-onset  16-39 years | Mid-onset  40-59 years | Late-onset  ≥60 years | *P*  (16-40 vs ≥60) | *P*  (40-59 vs ≥60) |
| --- | --- | --- | --- | --- | --- |
| Total, n (%) | 3728 (54.3) | 2114 (30.8) | 1019 (14.9) |  |  |
| Sex, n (%) |  |  |  | **<0.001** | 0.10 |
| Male | 1598 (48.3) | 980 (52.1) | 523 (55.5) |  |  |
| Female | 1711 (51.7) | 900 (47.9) | 420 (44.5) |  |  |
| Age at diagnosis, Median (IQR) | 27.0 (22.0-33.0) | 49 (44.0-54.0) | 66 (62.0-70.0) | **<0.001** | **<0.001** |
| Years since diagnosis, median (IQR) | 12.0 (10.0-13.0) | 12.0 (10.0-13.0) | 11 (10.0-13.0) | 0.12 | 0.085 |
| Smoking status, n (%) |  |  |  | **<0.001** | **<0.001** |
| Never smoker | 2233 (62.5) | 806 (39.4) | 327 (33.0) | **<0.001** | **0.003** |
| Current smoker | 215 (6.0) | 110 (5.4) | 28 (2.8) | **<0.001** | **0.005** |
| Former smoker | 1126 (31.5) | 1129 (55.2) | 637 (64.2) | **<0.001** | **<0.001** |
| BMI, median (IQR) | 24.7 (22.1-28.2) | 27.1 (24.4-30.8) | 26.8 (24.4-30.1) | **<0.001** | 0.072 |
| BMI range kg/m^2^, n (%) |  |  |  | **<0.001** | **0.002** |
| <18.5 | 46 (2.5) | 13 (1.0) | 6 (0.9) | **0.048** | 1 |
| 18.5-24.9 | 926 (50.2) | 362 (29.1) | 204 (31.4) | **<0.001** | 1 |
| 25-29.9 | 555 (30.1) | 498 (40.0) | 272 (41.9) | **<0.001** | 1 |
| >30 | 318 (17.2) | 373 (29.9) | 167 (25.7) | **<0.001** | **0.65** |
| IBD-related hospital admission | 1675 (47.9) | 658 (32.7) | 250 (26.1) | **<0.001** | **<0.001** |
| Any comorbidity, n (%) | 707 (19.0) | 855 (40.4) | 619 (60.7) | **<0.001** | **<0.001** |
| Hypertension | 62 (2.8) | 319 (22.0) | 335 (42.1) | **<0.001** | **<0.001** |
| Hypercholesterolaemia | 41 (1.9) | 262 (18.1) | 254 (32.0) | **<0.001** | **<0.001** |
| Diabetes | 30 (1.4) | 137 (9.4) | 136 (17.1) | **<0.001** | **<0.001** |
| Asthma | 353 (16.2) | 223 (15.3) | 119 (15.0) | 0.44 | 0.87 |
| COPD | 1 (0.1) | 7 (0.6) | 11 (1.5) | **<0.001** | **0.049** |
| Cerebrovascular disease | 5 (0.2) | 23 (1.6) | 31 (3.9) | **<0.001** | **<0.001** |
| Cardiovascular disease | 16 (0.7) | 88 (6.0) | 163 (20.5) | **<0.001** | **<0.001** |
| Coronary artery disease | 14 (0.6) | 68 (4.7) | 142 (17.9) | **<0.001** | **<0.001** |
| Myocardial infarction | 2 (0.1) | 29 (2.0) | 44 (5.5) | **<0.001** | **<0.001** |
| Congestive heart failure | 0 (0) | 5 (0.3) | 12 (1.5) | **<0.001** | **0.005** |
| Multiple sclerosis | 7 (0.2) | 4 (0.2) | 2 (0.2) | 1 | 1 |
| Arthritis | 82 (3.8) | 205 (14.1) | 162 (20.4) | **<0.001** | **<0.001** |
| Rheumatoid Arthritis | 41 (1.9) | 97 (6.7) | 62 (7.8) | **<0.001** | 0.39 |
| Lymphoma | 5 (0.2) | 8 (0.4) | 5 (0.5) | **<0.001** | 0.91 |
| Cancer | 242 (11.1) | 215 (14.8) | 176 (22.1) | **<0.001** | **<0.001** |
| Any EIM, n (%) | 351 (11.3) | 253 (14.1) | 114 (12.9) | 0.21 | 0.42 |
| Ankylosing spondylitis | 48 (1.5) | 43 (2.4) | 17 (1.9) | 0.56 | 0.50 |
| Enteropathic arthritis | 99 (3.2) | 92 (5.1) | 43 (4.8) | 0.024 | 0.81 |
| Psoriasis | 109 (3.5) | 89 (4.9) | 41 (4.5) | 0.18 | 0.75 |
| Erythema nodosum | 23 (0.7) | 12 (0.7) | 3 (0.3) | 0.27 | 0.42 |
| Iritis | 40 (1.3) | 41 (2.2) | 19 (2.1) | 0.10 | 0.90 |
| Orofacial granulomatosis | 12 (0.4) | 5 (0.3) | 0 (0) | 0.13 | 0.27 |
| Primary sclerosing cholangitis | 66 (2.1) | 21 (1.2) | 7 (0.8) | 0.012 | 0.46 |

**Supplementary Table 4. Sensitivity analysis of UC and CD disease phenotype in young-onset (16-39 years), mid-onset (40-59 years) and late-onset (≥60 years) IBD with longer disease duration. Disease duration was between 8-13 years in CD, and 9-15 years in UC.**

|  |  | Young-onset  16-39 years | Mid-onset  40-59 years | Late-onset  ≥60 years | *P*  16-39 vs ≥60 | *P*  40-59 vs ≥60 |
| --- | --- | --- | --- | --- | --- | --- |
|  | Ulcerative colitis | | | | | |
|  | Total, n (%) | 3728 (54.3) | 2113 (30.8) | 1019 (14.9) |  |  |
|  | UC disease extent |  |  |  | **0.002** | **<0.001** |
|  | E1 (proctitis) | 594 (17.9) | 393 (20.7) | 134 (14.6) | 0.09 | **<0.001** |
|  | E2 (left-sided) | 1612 (48.6) | 981 (51.8) | 504 (55.0) | **0.006** | 0.34 |
|  | E3 (extensive) | 1112 (33.5) | 520 (27.5) | 279 (30.4) | 0.23 | 0.34 |
|  |  |  |  |  |  |  |
| Crohn’s disease | | | | | | |
| Total, n (%) | | 3406 (61.6) | 1457 (26.4) | 664 (12.0) |  |  |
| CD disease location | |  |  |  | **<0.001** | **0.028** |
| L1 (ileal) | | 1311 (39.9) | 700 (49.0) | 292 (45.4) | **0.035** | **0.50** |
| L2 (colonic) | | 834 (25.4) | 421 (29.5) | 224 (34.8) | **<0.001** | 0.17 |
| L3 (ileocolonic) | | 1106 (33.6) | 293 (20.5) | 115 (17.9) | **<0.001** | 0.50 |
| L4 (upper gastrointestinal) | | 38 (1.2) | 15 (1.0) | 12 (1.9) | **0.42** | **0.50** |
| CD disease behavior | |  |  |  | **<0.001** | **0.068** |
| Inflammatory | | 2264 (70.3) | 989 (72.0) | 455 (72.7) | 0.70 | 1.0 |
| Stricturing | | 595 (18.5) | 275 (20.0) | 138 (22.0) | **<0.001** | 1.0 |
| Penetrating | | 361 (11.2) | 110 (8.0) | 33 (5.3) | **<0.001** | 0.25 |
| Perianal disease | | 998 (31.3) | 290 (21.2) | 94 (14.9) | **<0.001** | **0.001** |
| Tags/fissures/ulcers | | 392 (11.5) | 122 (8.4) | 33 (5.0) | **<0.001** | **0.007** |
| Perianal abscess | | 425 (12.5) | 112 (7.7) | 34 (5.1) | **<0.001** | **0.038** |
| Simple fistula | | 315 (9.2) | 85 (5.8) | 27 (4.1) | **<0.001** | 0.113 |
| Complex fistula | | 212 (6.2) | 51 (3.5) | 19 (2.9) | **0.001** | 0.527 |

**Supplementary Table 5. Proportionate use of IBD-specific medication and surgery in unmatched ulcerative colitis and Crohn’s disease.**

|  | Young-onset  16-39 years | Mid-onset  40-59 years | Late-onset  ≥60 years | *P*  16-39 vs ≥60 | *P*  40-59 vs ≥60 |
| --- | --- | --- | --- | --- | --- |
| Ulcerative colitis |  |  |  |  |  |
| Total, n (%) | 9480 (56.0) | 5203 (30.7) | 2247 (13.3) |  |  |
| 5-ASA |  |  |  |  |  |
| All-time | 7738 (81.6) | 4429 (81.3) | 1834 (81.6) | 1 | 0.75 |
| 2-yr | 5890 (66.6) | 3252 (66.1) | 1520 (70.8) | **<0.001** | **<0.001** |
| 5-yr | 6303 (71.3) | 3503 (71.2) | 1618 (75.4) | **<0.001** | **<0.001** |
| Corticosteroid |  |  |  |  |  |
| All-time | 6100 (64.3) | 2939 (56.5) | 1187 (52.8) | **<0.001** | **0.004** |
| 2-yr | 3605 (39.9) | 1729 (34.4) | 789 (36.2) | **0.002** | **0.13** |
| 5-yr | 4325 (47.9) | 2082 (41.4) | 957 (44.0) | **0.001** | **0.044** |
| Immunomodulator |  |  |  |  |  |
| All-time | 4829 (50.9) | 2184 (42.0) | 672 (29.9) | **<0.001** | **<0.001** |
| 2-yr | 2808 (29.7) | 1225 (23.6) | 416 (18.5) | **<0.001** | **<0.001** |
| 5-yr | 3681 (38.9) | 1630 (31.4) | 543 (24.2) | **<0.001** | **<0.001** |
| Anti-TNF |  |  |  |  |  |
| All-time | 3515 (37.1) | 1306 (25.1) | 391 (17.4) | **<0.001** | **<0.001** |
| 2-yr | 1460 (19.4) | 562 (13.9) | 213 (10.4) | **<0.001** | **<0.001** |
| 5-yr | 2197 (29.2) | 849 (21.0) | 317 (15.4) | **<0.001** | **<0.001** |
| Anti-TNF & immunomodulator |  |  |  |  |  |
| All-time | 2224 (24.6) | 813 (16.2) | 191 (8.7) | **<0.001** | **<0.001** |
| 2-yr | 920 (9.7) | 378 (7.3) | 118 (5.3) | **<0.001** | **0.002** |
| 5-yr | 1506 (15.9) | 594 (11.4) | 189 (8.4) | **<0.001** | **<0.001** |
| Vedolizumab |  |  |  |  |  |
| All-time | 1357 (14.3) | 644 (12.4) | 349 (15.5) | 0.15 | **<0.001** |
| 2-yr | 318 (7.5) | 128 (5.8) | 131 (10.3) | 0.002 | **<0.001** |
| 5-yr | 546 (12.8) | 246 (11.2) | 212 (16.6) | 0.001 | **<0.001** |
| Colectomy |  |  |  |  |  |
| All-time | 432 (4.8) | 184 (3.7) | 83 (3.9) | 0.077 | 0.82 |
| 2-yr | 206 (2.3) | 97 (2.0) | 58 (2.7) | 0.28 | 0.061 |
| 5-yr | 300 (3.3) | 66 (3.1) | 66 (3.1) | 0.61 | 0.56 |
|  | **Young-onset**  **16-39 years** | **Mid-onset**  **40-59 years** | **Late-onset**  **≥60 years** | ***P***  **16-39 vs ≥60** | ***P***  **40-59 vs ≥60** |
| Crohn’s Disease |  |  |  |  |  |
| Total, n (%) | 9921 (65.8) | 3752 (24.9) | 1409 (9.3) |  |  |
| 5-ASA |  |  |  |  |  |
| All-time | 3441 (34.7) | 1318 (35.1) | 501 (35.6) | 0.54 | 0.80 |
| 2-yr | 2568 (26.6) | 1022 (27.8) | 399 (29.0) | 0.062 | 0.42 |
| 5-yr | 2769 (28.6) | 1114 (30.3) | 421 (30.6) | 0.15 | 0.87 |
| Corticosteroid |  |  |  |  |  |
| All-time | 6289 (63.4) | 2208 (58.8) | 807 (57.3) | **<0.001** | 0.32 |
| 2-yr | 3968 (42.1) | 1470 (40.7) | 567 (41.8) | **0.84** | 0.52 |
| 5-yr | 4498 (47.7) | 1685 (46.7) | 652 (48.0) | **0.86** | 0.41 |
| Immunomodulator |  |  |  |  |  |
| All-time | 7413 (74.7) | 2426 (64.7) | 644 (45.7) | **<0.001** | **<0.001** |
| 2-yr | 4957 (50.3) | 1636 (43.8) | 461 (32.7) | **<0.001** | **<0.001** |
| 5-yr | 5817 (59.0) | 1950 (52.2) | 552 (39.2) | **<0.001** | **<0.001** |
| Anti-TNF |  |  |  |  |  |
| All-time | 6483 (65.3) | 1861 (49.6) | 502 (35.6) | **<0.001** | **<0.001** |
| 2-yr | 3191 (32.4) | 1015 (27.2) | 308 (21.9) | **<0.001** | **<0.001** |
| 5-yr | 4320 (43.9) | 1374 (36.8) | 418 (29.7) | **<0.001** | **<0.001** |
| Anti-TNF & immunomodulator |  |  |  |  |  |
| All-time | 4365 (48.9) | 1134 (32.5) | 243 (18.1) | **<0.001** | **<0.001** |
| 2-yr | 2254 (22.8) | 639 (17.0) | 158 (11.2) | **<0.001** | **<0.001** |
| 5-yr | 3247 (32.8) | 939 (25.1) | 234 (16.6) | **<0.001** | **<0.001** |
| Vedolizumab |  |  |  |  |  |
| All-time | 800 (8.1) | 337 (9.0) | 226 (16.0) | **<0.001** | **<0.001** |
| 2-yr | 94 (2.4) | 72 (4.1) | 98 (11.3) | **<0.001** | **<0.001** |
| 5-yr | 172 (4.4) | 115 (6.6) | 147 (16.9) | **<0.001** | **<0.001** |
| Ustekinumab |  |  |  |  |  |
| All-time | 757 (7.6) | 261 (7.0) | 96 (6.8) | **0.30** | **0.91** |
| 2-yr | 91 (3.4) | 46 (3.9) | 43 (7.2) | **<0.001** | **0.004** |
| 5-yr | 194 (7.3) | 92 (7.8) | 64 (10.7) | **0.007** | **0.056** |
| Intestinal resection |  |  |  |  |  |
| All-time | 2517 (26.3) | 824 (22.6) | 235 (17.3) | **<0.001** | **<0.001** |
| 2-yr | 1398 (14.8) | 546 (15.2) | 168 (12.5) | **0.024** | **0.015** |
| 5-yr | 1816 (19.3) | 654 (18.3) | 200 (14.8) | **<0.001** | **0.005** |
| Perianal surgery |  |  |  |  |  |
| All-time | 1126 (12.8) | 223 (6.1) | 51 (3.8) | **<0.001** | **0.001** |
| 2-yr | 562 (5.9) | 110 (3.0) | 25 (1.8) | **<0.001** | **0.027** |
| 5-yr | 742 (7.8) | 142 (3.9) | 32 (2.4) | **<0.001** | **0.010** |

N (%) refers to the total number and proportion of patients exposed to each outcome within the whole unmatched age cohorts.

**Supplementary Table 6. Log-rank tests comparing time to surgery in young-onset (16-39 years), mid-onset (40-59 years) and late-onset (≥60 years) UC and CD.**

|  |  | Log rank test *P* |
| --- | --- | --- |
| Colectomy in UC | YO vs MO | 0.52 |
|  | YO vs LO | 0.33 |
|  | MO vs LO | 0.77 |
| Intestinal resection in CD | YO vs MO | 0.29 |
|  | YO vs LO | 0.29 |
|  | MO vs LO | 0.94 |
| Perianal surgery in perianal CD | YO vs MO | 0.63 |
|  | YO vs LO | **0.005** |
|  | MO vs LO | 0.03 |

**Supplementary Table 7. Sensitivity analysis showing indication for colectomy in young-onset, mid-onset and late-onset ulcerative colitis in patients with longer disease duration (9-15 years).**

|  | Young-onset  16-39 years | Mid-onset  40-59 years | Late-onset  ≥60 years | *P*  16-39 vs ≥60 | *P*  40-59 vs ≥60 |
| --- | --- | --- | --- | --- | --- |
| Colectomy indication, n (%) |  |  |  | **0.003** | 0.099 |
| Acute severe UC | 95 (54.9) | 35 (42.7) | 19 (46.3) | 1 | 1 |
| Chronic continuous colitis | 43 (24.9) | 21 (25.6) | 8 (19.5) | **0.045** | 1 |
| Colorectal cancer | 2 (1.2) | 4 (4.9) | 4 (9.8) | **0.041** | 1 |

**Supplementary Table 8. Sensitivity analysis showing demographic characteristics in young-onset (16-39 years), mid-onset (40-59 years) and late-onset (≥60 years) Crohn’s disease with no difference in disease duration (between 8-13 years in all groups).**

|  | Young-onset  16-39 years | Mid-onset  40-59 years | Late-onset  ≥60 years | *P*  (16-39 vs ≥60) | *P*  (40-59 vs ≥60) |
| --- | --- | --- | --- | --- | --- |
| Total, n (%) | 3406 (61.6) | 1457 (26.4) | 664 (12.0) |  |  |
| Sex, n (%) |  |  |  | **0.97** | **0.044** |
| Male | 1427 (47.2) | 573 (44.0) | 283 (47.0) |  |  |
| Female | 1597 (52.8) | 728 (56.0) | 319 (53.0) |  |  |
| Age at diagnosis, Median (IQR) | 25.0 (20.0-30.0) | 49.0 (44.0-53.0) | 66.0 (63.0-71.0) | **<0.001** | **<0.001** |
| Years since diagnosis, Median (IQR) | 10.0 (9.0-12.0) | 10.0 (9.0-12.0) | 10.0 (9.0-12.0) | **0.083** | **<0.001** |
| Smoking status, n (%) |  |  |  | **<0.001** | **<0.001** |
| Never smoker | 1833 (55.7) | 520 (36.6) | 211 (32.6) | **<0.001** | **<0.001** |
| Current smoker | 550 (16.7) | 289 (20.4) | 82 (12.7) | **<0.001** | **<0.001** |
| Former smoker | 908 (27.6) | 611 (43.0) | 354 (54.7) | **<0.001** | **<0.001** |
| BMI, Median (IQR) | 25.0 (22.0-29.1) | 27.5 (24.1-31.7) | 26.2 (23.1-29.6) | **<0.001** | **<0.001** |
| BMI range kg/m^2^, n (%) |  |  |  | **<0.001** | **<0.001** |
| <18.5 | 50 (3.1) | 13 (1.5) | 14 (3.3) | 1 | 0.22 |
| 18.5-24.9 | 752 (47.0) | 273 (31.7) | 154 (36.0) | **<0.001** | 0.36 |
| 25-29.9 | 455 (28.5) | 287 (33.3) | 163 (38.1) | **<0.001** | 0.36 |
| >30 | 342 (21.4) | 289 (33.5) | 97 (22.7) | 1 | **0.007** |
| IBD-related hospital admission | 1794 (56.0) | 583 (41.8) | 243 (39.4) | **<0.001** | **<0.001** |
| Any comorbidity, n (%) | 668 (19.6) | 545 (37.4) | 402 (60.5) | **<0.001** | **<0.001** |
| Hypertension | 43 (2.2) | 209 (21.2) | 209 (41.5) | **<0.001** | **<0.001** |
| Hypercholesterolaemia | 23 (1.2) | 135 (13.7) | 141 (28.1) | **<0.001** | **<0.001** |
| Diabetes | 30 (1.6) | 80 (8.1) | 81 (16.0) | **<0.001** | **<0.001** |
| Asthma | 349 (18.2) | 163 (16.5) | 83 (16.4) | 0.39 | 0.91 |
| COPD | 1 (0.1) | 16 (2.0) | 12 (2.7) | **<0.001** | **<0.001** |
| Cerebrovascular disease | 4 (0.2) | 15 (1.5) | 23 (4.6) | **<0.001** | **<0.001** |
| Cardiovascular disease | 15 (0.8) | 44 (4.5) | 88 (17.6) | **<0.001** | **<0.001** |
| Coronary artery disease | 12 (0.6) | 35 (3.5) | 71 (14.1) | **<0.001** | **<0.001** |
| Myocardial infarction | 0 (0) | 9 (0.9) | 28 (5.5) | **<0.001** | **<0.001** |
| Congestive heart failure | 0 (0) | 4 (0.4) | 6 (1.2) | **<0.001** | **<0.001** |
| Multiple sclerosis | 5 (0.2) | 3 (0.2) | 3 (0.5) | 0.26 | 0.44 |
| Arthritis | 108 (5.6) | 133 (13.4) | 114 (22.6) | **<0.001** | **<0.001** |
| Rheumatoid Arthritis | 57 (3.0) | 58 (5.9) | 40 (7.9) | **<0.001** | 0.13 |
| Lymphoma | 4 (0.1) | 7 (0.6) | 5 (0.9) | **0.007** | 0.12 |
| Cancer | 246 (12.8) | 138 (14.0) | 127 (25.1) | **<0.001** | **<0.001** |
| Any EIM, n (%) | 541 (18.7) | 264 (21.0) | 108 (18.8) | 0.97 | 0.57 |
| Ankylosing spondylitis | 54 (1.9) | 51 (4.0) | 26 (4.5) | **<0.001** | 0.82 |
| Enteropathic arthritis | 171 (5.9) | 90 (7.2) | 39 (6.8) | **0.45** | 0.83 |
| Psoriasis | 194 (6.7) | 86 (6.8) | 36 (6.2) | 0.76 | 0.79 |
| Erythema nodosum | 98 (3.4) | 24 (1.9) | 6 (1.0) | 0.004 | 1 |
| Iritis | 75 (2.6) | 48 (3.8) | 18 (3.1) | 0.56 | **0.037** |
| Orofacial granulomatosis | 74 (2.6) | 19 (1.5) | 10 (1.7) | 0.30 | 0.11 |
| Primary sclerosing cholangitis | 19 (0.7) | 9 (0.7) | 6 (1.0) | 0.46 | 0.85 |

**Supplementary Table 9. Adjusted OR for medication use in propensity score-matched patients with young-onset (16-39 years), mid-onset (40-59 years), and late-onset (≥60 years) with complex fistulizing perianal Crohn’s disease.**

| Complex Fistulizing Perianal Crohn’s disease | **Age group** | **No**  **n (%)** | **Yes**  **n (%)** | **OR multivariable**  **(95% CI)** | **P** |
| --- | --- | --- | --- | --- | --- |
| 5-ASA | Young-onset | 37 (57.8) | 27 (42.2) | 0.80 (0.33-1.95) | 0.63 |
|  | Mid-onset | 34 (59.6) | 23 (40.4) | 0.96 (0.43-2.12) | 0.92 |
|  | Late-onset | 43 (58.1) | 31 (41.9) | - |  |
| Corticosteroids | Young-onset | 27 (42.2) | 37 (57.8) | 1.67 (0.71-3.98) | 0.24 |
|  | Mid-onset | 28 (49.1) | 29 (50.9) | 1.28 (0.60-2.76) | 0.53 |
|  | Late-onset | 38 (51.4) | 36 (48.6) | - | - |
| Immunomodulator | Young-onset | 8 (12.5) | 56 (87.5) | 12.36 (3.78-47.62) | **<0.001** |
|  | Mid-onset | 16 (28.1) | 41 (71.9) | 3.40 (1.37-9.11) | **0.011** |
|  | Late-onset | 34 (45.9) | 40 (54.1) | - |  |
| Anti-TNF | Young-onset | 9 (14.1) | 55 (85.9) | 5.75 (1.99-18.21) | **0.002** |
|  | Mid-onset | 16 (28.1) | 41 (71.9) | 1.88 (0.80-4.53) | 0.15 |
|  | Late-onset | 28 (37.8) | 46 (62.2) | - | - |
| Anti-TNF & immunomodulator | Young-onset | 14 (23.7) | 45 (76.3) | 12.64 (4.48-39.68) | **<0.001** |
|  | Mid-onset | 26 (47.3) | 29 (52.7) | 3.01 (1.31-7.17) | **0.01** |
|  | Late-onset | 45 (65.2) | 24 (34.8) | - | - |

**Supplementary Table 10. Cox proportional hazards regression analysis between propensity score-matched cohorts comparing risk of perianal surgery in complex fistulizing perianal CD.**

|  |  | N (%) | HR (multivariable)  (95% CI) | P |
| --- | --- | --- | --- | --- |
| Perianal surgery in complex fistulizing perianal CD | Young-onset | 55 (31.6) | 2.37 (1.27-4.41) | **0.007** |
|  | Mid-onset | 52 (29.9) | 1.84 (1.01-3.36) | **0.047** |
|  | Late-onset | 67 (38.5) | - | - |

**Supplementary Figure 1. Consort diagram showing patient eligibility and inclusion**

33,114 identified

1102 with IBD-U excluded

32,012

UC – 16,930

Age group:

16-39 - 9480

40-59 - 5203

≥ 60 − 2247

CD – 15,082

Age group:

16-39 - 9921

40-59 - 3752

≥ 60 − 1409

**Supplementary Figure 2. Time trend analysis of IBD-related medications in UC and CD patients from 2000-2025. For each year, the proportionate use of each drug class (as a percentage) was calculated from the drug start dates. Panel A: results for young-onset UC (16-39 years). Panel B: results for mid-onset UC (40-59 years). Panel C: results for late-onset UC (≥60 years). Panel D: results for young-onset CD (16-39 years). Panel E: results for mid-onset CD (40-59 years). Panel F: results for late-onset CD (≥60 years).**

F
